# Examining functional group-dependent effects on the ionization of lignin monomers using supercritical fluid chromatography/electrospray ionization mass spectrometry

**DOI:** 10.1007/s00216-024-05358-x

**Published:** 2024-06-03

**Authors:** Jens Prothmann, Daniel Molins-Delgado, Alexander Braune, Margareta Sandahl, Charlotta Turner, Peter Spégel

**Affiliations:** Centre for Analysis and Synthesis, Department of Chemistry, Lund University, P.O. Box 124, SE-22100 Lund, Sweden

**Keywords:** Design of experiments, Retention prediction, Electrospray ionization efficiency, Triple quadrupole mass spectrometry, Molecular descriptors

## Abstract

The chemical and biological conversion of biomass-derived lignin is a promising pathway for producing valuable low molecular weight aromatic chemicals, such as vanillin or guaiacol, known as lignin monomers (LMs). Various methods employing chromatography and electrospray ionization-mass spectrometry (ESI–MS) have been developed for LM analysis, but the impact of LM chemical properties on analytical performance remains unclear. This study systematically optimized ESI efficiency for 24 selected LMs, categorized by functionality. Fractional factorial designs were employed for each LM to assess ESI parameter effects on ionization efficiency using ultra-high-performance supercritical fluid chromatography/ESI–MS (UHPSFC/ESI–MS). Molecular descriptors were also investigated to explain variations in ESI parameter responses and chromatographic retention among the LMs. Structural differences among LMs led to complex optimal ESI settings. Notably, LMs with two methoxy groups benefited from higher gas and sheath gas temperatures, likely due to their lower log *P* and higher desolvation energy requirements. Similarly, vinyl acids and ketones showed advantages at elevated gas temperatures. The retention in UHPSFC using a diol stationary phase was correlated with the number of hydrogen bond donors. In summary, this study elucidates structural features influencing chromatographic retention and ESI efficiency in LMs. The findings can aid in developing analytical methods for specific technical lignins. However, the absence of an adequate number of LM standards limits the prediction of LM structures solely based on ESI performance data.

**Graphical Abstract:**

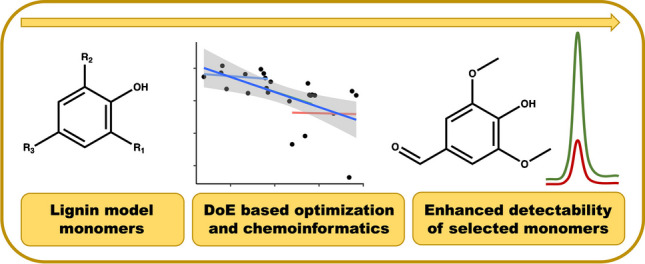

**Supplementary Information:**

The online version contains supplementary material available at 10.1007/s00216-024-05358-x.

## Introduction

**Fig. 1:**
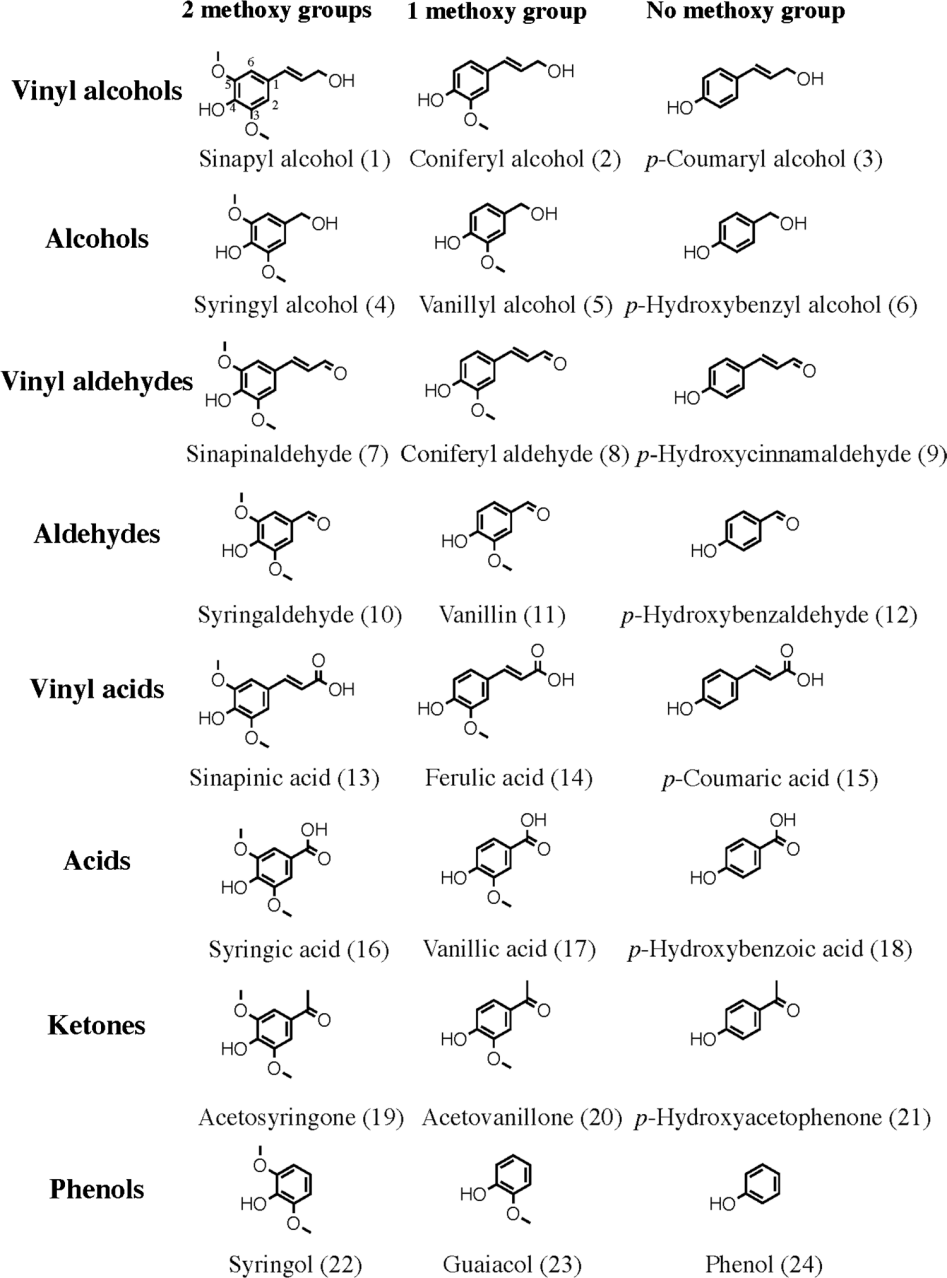
Selected lignin monomer model compounds categorized into groups based on functionality and the degree of methoxylation

Native lignin primarily comprises three monomeric phenylpropanoid units: *p-*coumaryl alcohol (H-unit), coniferyl alcohol (G-unit), and sinapyl alcohol (S-unit), differing only in the number of methoxy groups (Fig. 1). Within the polymeric structure of native lignin, these units are linked by various ether or carbon–carbon bonds.

The isolation of native lignin from biomass involves different processes, with the kraft and sulphite processes being the most common [1, 2]. Throughout the isolation, the lignin biopolymer undergoes degradation, forming new functional groups through bond cleavage and re-condensation reactions, resulting in what is termed technical lignin [2, 3]. Further chemical conversion of technical lignin into low molecular weight aromatic chemicals introduces additional functional groups [1], such as alcohols, aldehydes, ketones, ethers, and carboxylic acids [2, 4–6]. Consequently, numerous mono-aromatic compounds, collectively known as lignin monomers (LMs), are generated from the isolation and conversion of lignin, thereby increasing the chemical complexity of the resulting mixtures.

Industries in the biofuel, food, and pharmaceutical sectors display interest in specific LMs like vanillin and syringaldehyde [7–9]. The intricate chemical compositions of converted technical lignins pose a significant challenge in valorising LMs [10, 11]. Consequently, there is a need for selective, qualitative, and quantitative analytical methods to analyse LMs within complex lignin samples.

Over the years, gas chromatography (GC) [11–14], liquid chromatography (LC) [15, 16], and supercritical fluid chromatography (SFC) [17–19], coupled with mass spectrometry (MS), have been employed for LM analysis [20]. One primary advantage of GC/MS utilizing electron ionization (EI), compared to LC/MS and SFC/MS utilizing atmospheric pressure ionization (API), lies in its wider ionization selectivity [15]. However, recent advancements have enhanced ionization efficiency in API for both LC/MS [16] and SFC/MS [17, 18].

In comparison to EI, API can yield high-intensity molecular ions, subsequently fragmented under controlled conditions. For instance, Owen et al*.* developed a high-performance liquid chromatography (HPLC)/high-resolution mass spectrometry (HRMS) method employing electrospray ionization (ESI) in negative mode for LM analysis in a depolymerized lignin sample [16]. They improved ionization efficiency by introducing a sodium hydroxide solution post-HPLC column using a tee connector [16]. Our group recently published an ultra-high-performance supercritical fluid chromatography (UHPSFC)/HRMS method using ESI in negative mode for LMs [17], which has been applied to various lignin samples [21, 22]. Systematic optimization of ESI efficiency was achieved by screening nine SFC/ESI–MS variables employing an interaction model with a D-optimal design [17].

However, both studies aimed at enhancing the overall ionization efficiency of complex LM mixtures. Therefore, while these methods provide relatively good ESI efficiency for most LMs, they may not offer optimal conditions for individual LMs. Consistent with this notion, Andrianova et al. demonstrated that structural variations and the presence of hydroxyl, methoxy, and carboxyl groups significantly influence the ESI process [23]. Hence, optimizing ESI efficiency separately for each functional group could aid in developing tailored UHPSFC/ESI–MS methods for specific LMs of interest or particular lignin samples. To our knowledge, no systematic functional group-based optimization of ESI efficiency for LMs has been reported to date.

In this study, we present a systematic approach for functional group-based optimization of ESI efficiency for 24 selected LMs across 8 structural classes, using UHPSFC/ESI-triple quadrupole mass spectrometry (QQQ).

## Materials and methods

### Chemicals

Acetosyringone, acetovanillone, coniferyl alcohol, coniferyl aldehyde, *p-*coumaric acid, *p-*coumaryl alcohol, ferulic acid, guaiacol, *p-*hydroxy-acetophenone, *p-*hydroxybenz-aldehyde, *p-*hydroxybenzoic acid, *p-*hydroxybenzyl alcohol, phenol, sinapinic acid, sinapinaldehyde, syringaldehyde, syringol, syringic acid, sinapyl alcohol, vanillic acid, vanillin, and vanillyl alcohol were purchased from Sigma-Aldrich (St. Louis, MO, USA). Syringyl alcohol was obtained from Alfa Aesar (Haverhill, MA, USA) and *p-*hydroxycinnamaldehyde from Combi-Blocks (San Diego, CA, USA). Ammonia (2 M solution in methanol) was purchased from Fisher Scientific (Waltham, MA, USA). Methanol (LC/MS grade) was purchased from Fisher Scientific (Pittsburgh, PA, USA), ethyl acetate (LC/MS grade) from Merck (Darmstadt, Germany), and CO_2_ (scientific grade, 5.2) from Linde (Munich, Germany). Stock solutions of all analytes (Fig. 1) were made in ethyl acetate with a concentration of 0.5 mg/mL. Working solution was prepared from these stock solutions by dilution in ethyl acetate, to provide a signal-to-noise ratio above 10 and well below the limit of linearity (see Electronic Supplementary Material Table S1).

### Equipment

An Agilent 1260 Infinity II SFC system equipped with a 1260 Infinity II SFC Control Module, a 1260 Infinity II isocratic pump, a 1260 Infinity II diode array detector, a 1260 Infinity II column compartment, a 1260 Infinity II SFC Multisampler, and a 1260 Infinity II SFC binary pump (Agilent Technologies, Santa Clara, CA, USA) connected to an Agilent 6495 triple quadrupole LC/MS system with an Agilent Jet Stream ESI source was used for all experiments. Separations were conducted on a Torus DIOL column (3 mm × 100 mm, 1.7 µm) equipped with a Torus DIOL VanGuard pre-column (2.1 mm × 5 mm, 1.7 µm), both from Waters (Milford, MA, USA).

### Software

The SFC/MS system was controlled, and data were evaluated using Agilent MassHunter 10.0. Experimental designs were created and assessed using ModdeTM 12.0.1 (Sartorius Stedim Biotech, Umeå, Sweden).

### Design of experiments

A model employing a fractional factorial design with a resolution of IV was utilized to explore the impact of seven quantitative variables on the ESI efficiency for each analyte individually. These seven variables comprised five ESI source parameters—capillary voltage, gas temperature, gas flow rate, sheath gas temperature, and sheath gas flow rate—and two variables associated with sample injection: feed speed and overfeed volume. Table 1 presents an overview of the investigated variables and their respective ranges.

**Table 1:** UHPSFC/MS variables screened and their respective ranges

Variables	Variable ranges		
	− 1	0	+ 1
SFC injection feed speed (µL/min)	200	400	600
SFC injection overfeed volume (µL)	2	5	8
ESI gas temperature (°C)	150	200	250
ESI sheath gas temperature (°C)	200	250	300
ESI capillary voltage (V)	2500	3000	3500
ESI gas flow (L/min)	12	14	16
ESI sheath gas flow (L/min)	9	10	11

Peak intensity served as the response variable, and a total of 19 experiments were conducted, encompassing three replicated centre points (see Electronic Supplementary Material Table S2). Multiple linear regression (MLR) was employed for model assessments, aiming to optimize models for the highest predictive capability (*R*^2^_pred_).

### UHPSFC/ESI–MS

UHPSFC parameters were based on a previous study [17], but with the important difference that elution was performed in isocratic mode, using a mobile phase consisting of CO_2_/methanol (90/10, v/v), to ensure identical chemical environment for all LMs during the ionization event. The injection volume amounted to 5 µL, and ethyl acetate served both as the injection flush solvent and the system wash solvent. The mobile phase flow rate was 1 mL/min and the column temperature was set to 50 °C. A backpressure of 130 bars was used, with a temperature of 60 °C on the backpressure regulator. A solvent containing 5 mM ammonia in methanol was used as the makeup solvent, to prevent precipitation of LMs and to enhance ionization, with a makeup solvent flow rate set at 0.3 mL/min. ESI was employed in negative mode, with a nebulizer pressure of 20 psi and a nozzle voltage of 1300 V. The QQQ was operated in single ion monitoring mode, targeting the molecular ions ([M-H]-) of each analyte. The MS cell accelerator voltage and the delta electron multiplier voltage were maintained at constant levels of 7 V and 400 V, respectively.

### Data handling and statistics

Due to the non-quantitative nature of the data, experimental results were normalized concerning the average of the centre point replicates for each investigated LM. The average retention times and peak intensities along with their corresponding repeatabilities for all LMs are shown in Electronic Supplementary Material Table S1.Molecular and electronic descriptors were computed from SMILES using the eval.desc function from the rCDK package. To streamline analysis, descriptors displaying negligible variance were identified using the nearZeroVar function from the caret package and subsequently eliminated. In instances where descriptors demonstrated high correlation (Spearman’s *r* > 0.8), only one of the correlated descriptors was retained. Linear models were constructed using the lm function from the stats package and optimized using the ols_step_all_possible function from the olsrr package. This optimization involved systematically removing variables to achieve the highest *R*^2^_pred_ while minimizing the number of variables used in the models. Predicted values were generated employing the predict function from the stats package.

## Results and discussion

**Fig. 2:**
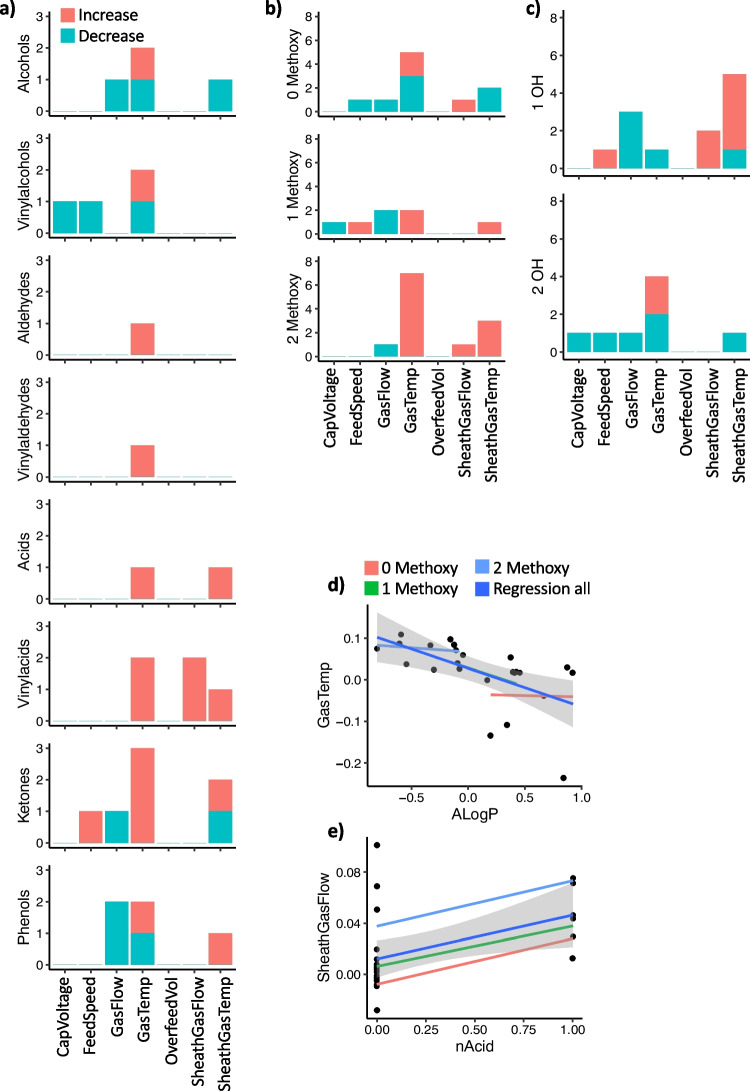
Impact of the investigated factors on the normalized signal intensity for the investigated LMs, divided into **a** functional category, **b** the number of methoxy groups on the phenol structure, and **c** the number of hydroxyl groups. Graphs are showing effects remaining after optimization of the models and their direction, i.e. the number of LMs within the assigned category that show increased or decreased signals when the setting of the indicated instrumental parameter is increased. **d** Variation in the response to changes in gas temperature (GasTemp) associated with the Ghose-Crippen log *k*_ow_ (AlogP). The plot shows how the effect of GasTemp depends on the AlogP of the LMs, indicating LMs with low AlogP to benefit from a higher GasTemp, whereas those with a higher AlogP tend to benefit from a lower GasTemp. The lines indicate regression for all LMs (Regression all; dark blue), and regression for LMs with 0 methoxy groups (red), 1 methoxy group (green), and 2 methoxy groups (light blue). The regression line for one methoxy group is hidden behind the line representing the overall regression (Regression all). **e** Variation in the response to alterations in sheath gas flow rate (SheathGasFlow) associated with the acidic group count (nAcid). LMs with higher nAcid benefit more from a higher SheathGasFlow. Lines are colored according to the number of methoxy groups as in panel **d**

Effects from the fractional factorial designs derived from the optimized linear models were characterized as either positive (yielding an increased signal) or negative (yielding a decreased signal) for the 8 different functionalities (Fig. 2a), 3 different degrees of methoxylation (Fig. 2b), and 2 different degrees of hydroxylation (Fig. 2c). Increasing gas temperature showed a positive effect on most LMs, except for alcohols, vinyl alcohols, and phenols (Fig. 2a). Notably, strong positive effects were observed for vinyl acids and ketones. Acids and vinyl acids particularly benefited from higher gas temperatures and sheath gas temperatures, suggesting a preference for higher desolvation energies, especially for LMs with relatively low pK_a_-values.

Diverse responses were noted when categorizing LMs based on the number of methoxy groups (Fig. 2b). For compounds lacking or with a single methoxy group, no consistent influence of the investigated variables was observed. However, for compounds with two methoxy groups, higher gas temperature significantly positively influenced seven out of eight compounds in the group. This trend might be due to the requirement for higher desolvation energy in this group, which exhibits slightly lower pKa and log *P* values than others (see Electronic Supplementary Material Table S3). These compounds display higher solubility and lower surface activity in methanol, necessitating higher desolvation energy to overcome solvation energy, as previously suggested by Kebarle and Verkerk [24]. When categorizing LMs based on the number of hydroxyl groups (Fig. 2c), no consistent influence of the investigated variables was observed, which is likely due to the clustering of a larger number of structurally dissimilar LMs when dividing all LMs into only two groups.

Overall, drawing general conclusions about differences in ESI performance between LMs was challenging due to variations in functionality and the number of methoxy groups. Subsequently, we aimed to describe how properties of the entire LM sample influenced their response to changes in ionization parameters. A total of 8 electronic and 17 constitutional descriptors were generated from SMILES, resulting in 19 variables after eliminating those with negligible variation. After removing correlated variables (Spearman, *r* > 0.8), this number reduced to 11 (Table 2). Several variables correlated with molecular weight, likely due to the selected analytes’ structural homogeneity.

**Table 2:** Electronic and molecular descriptors utilized for predicting alterations in response to variations in ESI parameters and retention time

Parameter	Description
Fsp3	Non-flatness of the molecule
XLogP	Log *P* based on atom-type method
MW	Molecular weight
nAtomP	Largest Pi system
nAtomLC	Largest chain
ALogP	Ghose-Crippen log *K*_ow_
AMR	Molar refractivity
nAcid	Acidic group count
tpsaEfficiency	Polar surface area relative molecular size
nHBAcc	H-bond acceptor count
nHBDon	H-bond donor count

Valid models were established for gas temperature and sheath gas flow rate (*R*^2^_pred_ > 0.7); however, the predictive ability of models for other variables was poor. For gas temperature, a model consisting of 5 terms provided *R*^2^ = 0.84, adjusted *R*^2^ (*R*^2^_adj_) = 0.79, and *R*^2^_pred_ = 0.72, with Ghose-Crippen log *K*_ow_ (ALogP) being the most influential parameter (*p* = 5.0e − 6). The linear relation between ALogP and the response to gas temperature changes supports the hypothesis that compounds with two methoxy groups necessitate higher desolvation energies due to lower Log *P* values (Fig. 2d). The model for sheath gas flow rate included 6 parameters and provided *R*^2^ = 0.89, *R*^2^_adj_ = 0.83, and *R*^2^_pred_ = 0.75. All model terms were highly significant (*p* < 2e − 5), with acidic group count (nAcid) displaying the most significant effect (*p* = 3.2e − 7) (Fig. 2e), consistent with the group-wise investigation of LMs (Fig. 2a).

Feed speed and overfeed volume, parameters accessible in Agilent SFC autosamplers, were anticipated to significantly impact peak height, particularly in isocratic elution where sample zone focusing at the top of the column is less efficient. Higher feed speeds were expected to yield more focused injection plugs, resulting in narrower peaks and increased peak intensity. Similarly, a sufficiently high overfeed volume was assumed to ensure quantitative sample transfer to the column by introducing a solvent plug after the analyte zone. Surprisingly, both variables demonstrated low or non-significant importance for the measured signal intensity of LMs (Fig. 2a, b).

Subsequently, we explored whether the variation in ionization parameters could predict structural features in LMs. Due to the limited size of the functional group samples, these analyses were only attempted for the number of methoxy groups. However, the generated models were generally inadequate. The best model (*R*^2^ = 0.46, *R*^2^_adj_ = 0.41, and *R*^2^_pred_ = 0.36) included two terms (gas temperature and sheath gas temperature), with gas temperature exhibiting the most influence (*p* = 0.0088; supplementary information Fig. S1a). Sheath gas temperature showed borderline significance (*p* = 0.058; see Electronic Supplementary Material Fig. S1b). Adjusting the models for functional groups had minimal impact, likely due to the limited statistical power in this analysis. While the developed model provided some indications regarding the number of methoxy groups in unknown LMs (see Electronic Supplementary Material Fig. S1c), a larger training set is likely necessary to construct useful models for structural prediction based on ESI performance.

**Fig. 3:**
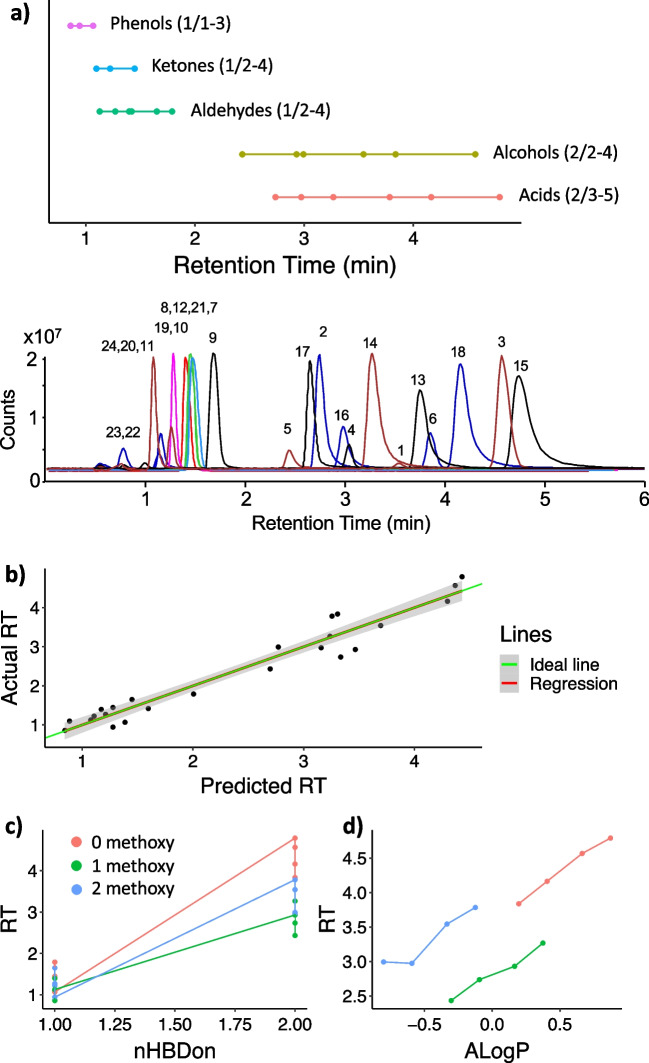
Prediction of LM retention times. **a** Retention time windows of the structural feature groups of the investigated LMs together with an annotated chromatogram (see numbers in Fig. 1). The number within parentheses indicates the number of H-bond donors/number of H-bond acceptors. **b** Actual versus predicted retention time (RT) from a model including log *P* based on the atom-type method (XLogP), acidic group count (nAcid), polar surface area relative molecular size (tpsaEfficiency), H-bond donor count (nHBDon), and Ghose-Crippen log *K*_ow_ (ALogP) for the investigated LMs. **c** RT as a function of nHBDon, and **d** as a function of ALogP. For clarity, only LMs with two hydrogen bond donors are displayed. Data are color-coded based on the number of methoxy groups in **c** and **d**

The retention time windows of the various investigated structural feature groups are depicted in Fig. 3a. Notably, alcohols and acids, all with H-bond donor count (nHBDon) = 2, exhibited longer retention on the DIOL stationary phase compared to other structural feature groups, all with nHBDon = 1. Additionally, the hydrogen bond acceptor count (nHBAcc) influenced retention, increasing within the structural feature groups with a rise in methoxy group counts (see Electronic Supplementary Material Table S3). Similar to nHBDon, a higher range of nHBAcc led to stronger hydrogen bonding interactions and subsequently higher retention. Finally, to verify the relevance of the molecular descriptors representing LM features, a linear model describing LM retention time was attempted. The optimized model included log *P* based on atom-type method (XLogP), nAcid, polar surface area relative molecular size (tpsaEfficiency), nHBDon, and ALogP (*R*^2^ = 0.94, *R*^2^_adj_ = 0.93, and *R*^2^_pred_ = 0.90) (Fig. 3b). The most influential parameter was nHBDon (*p* = 1.87e − 10), followed by ALogP (*p* = 4.30e − 5). Among LMs possessing a higher number of hydrogen donors, stronger hydrogen bonding interactions are anticipated with the diol stationary phase. Nevertheless, this effect varied based on the quantity of methoxy groups present on the LMs (Fig. 3c). The distinction observed can be predominantly explained by their differences in log *P* (Fig. 3d).

## Conclusions

The impact of ESI parameter settings on ionization yield for LMs is significantly influenced by the presence of functional groups and the number of methoxy groups within the LMs. Notably, higher gas and sheath gas temperatures proved advantageous for LMs exhibiting a high degree of methoxylation. This trend suggests a higher desolvation temperature necessity for LMs with lower log *P* values, which could be particularly relevant in studies concerning hardwood lignin rich in S-unit content. Additionally, vinyl acids and ketones demonstrated improved performance with elevated gas temperatures, although no distinct pattern was observed for other investigated functionalities. Although our results are based on quite general SFC conditions, we cannot rule out that changes in mobile phase and makeup fluid composition may impact on these results.

Despite the study’s limitations in predicting LM structural features from responses to ESI parameter variations, a model with relatively moderate precision was established for predicting the number of methoxy groups. In contrast, a robust model predicting retention times in SFC using a diol column was developed, emphasizing the significant role of hydrogen bonding capacity in determining retention.

In summary, our study strongly suggests that the structural characteristics of LMs play a pivotal role in determining both retention and ESI yield within the context of UHPSFC/QQQ-MS. These findings pave the way for further exploration of LM structural features to elucidate their impact on analytical outcomes. While the applicability of the presented results for analysing larger oligomers in technical lignin remains uncertain, the methodology developed here, along with expanded sets of oligomer chemical libraries, may pave the way for enhanced analytical strategies for larger molecular weight oligomers.

### Supplementary Information

Below is the link to the electronic supplementary material.

Supplementary file1 (DOCX 127 KB)
