# Supplementary material for: Examining functional group-dependent effects on the ionization of lignin monomers using supercritical fluid chromatography/electrospray ionization mass spectrometry
Source: Anal Bioanal Chem. 2024 Jun 3;416(18):4007–14. doi: 10.1007/s00216-024-05358-x (PMC11249424; doi:10.1007/s00216-024-05358-x)
Supplement: Supplementary file 1 — Supplementary file1 (DOCX 127 KB) [file 216_2024_5358_MOESM1_ESM.docx]

Supplementary Material

**Examining functional group-dependent effects on the ionization of lignin monomers using supercritical fluid chromatography/electrospray ionization mass spectrometry**

Jens Prothmann, Daniel Molins-Delgado, Alexander Braune, Margareta Sandahl, Charlotta Turner, Peter Spégel

**Contents:**

**Table S1.** Concentration in the working solutions, average retention times, repeatability of the retention times, average peak intensities and repeatability of peak intensities of all investigated LMs

**Table S2.** Experiments conducted within the fractional factorial design.

**Table S3.** Lignin monomers along with their respective retention times (RT), number of methoxy groups (OCH3), and predicted electronic and constitutional descriptors: Fsp3 (non-flatness of the molecule), XLogP (log P based on atom-type method), MW (molecular weight), nAtomP (largest Pi system), nAtomLC (largest chain), ALogP (Ghose-Crippen log K_ow_), AMR (molar refractivity), nAcid (acidic group count), tpsaE (polar surface area relative molecular size), nHBDon (H-bond donor count). Also given are pKa-values for each lignin monomer. Parameters were estimated using rCDK, accessing the chemoinformatic functionality of the CDK java library.

**Figure S1.** Prediction of the number of methoxy groups from alterations in response to variation in ESI parameters. The optimal settings of gas temperature (A) and sheath gas temperature (B) varied depending on the number of methoxy groups present on the LMs. (C) A model comprising these two factors exhibited promising potential for predicting the number of methoxy groups on LMs.

**Table S1.**

| Compound | Concentration in µg/mL | Average RT in min (n = 3) | Repeatability as relative standard deviation of the RT in % (n=3) | Average peak intensity in min (n=3) | Repeatability as relative standard deviation of the peak intensity in % (n=3) |
| --- | --- | --- | --- | --- | --- |
| Vanillyl alcohol | 2.5 | 2.43 | 0.47 | 1563526 | 2.92 |
| Sinapyl alcohol | 2.5 | 3.54 | 0.33 | 370801 | 2.48 |
| *p*-Coumaryl alcohol | 2.5 | 4.57 | 0.51 | 10493110 | 1.82 |
| Syringol | 2.5 | 0.94 | 1.62 | 540367 | 12.86 |
| Sinapinaldehyde | 1.25 | 1.65 | 0.00 | 12413009 | 3.30 |
| Syringyl alcohol | 2.5 | 2.99 | 1.58 | 2886128 | 5.00 |
| Sinapinic acid | 2.5 | 3.78 | 2.40 | 8723867 | 6.16 |
| *p*-Coumaric acid | 2.5 | 4.79 | 2.35 | 12017438 | 3.37 |
| Acetovanillone | 2.5 | 1.10 | 0.53 | 1755475 | 6.33 |
| Syringic acid | 2.5 | 2.97 | 1.55 | 4835356 | 9.79 |
| *p*-Hydroxybenzoic acid | 2.5 | 4.16 | 1.32 | 9037120 | 9.29 |
| Vanillin | 2.5 | 1.13 | 0.51 | 2561430 | 1.84 |
| Vanillic acid | 2.5 | 2.74 | 0.56 | 7928165 | 6.52 |
| *p*-Hydroxybenzyl alcohol | 2.5 | 3.84 | 0.30 | 2616610 | 3.55 |
| Acetosyringone | 2.5 | 1.22 | 0.94 | 4582046 | 9.42 |
| Ferulic acid | 2.5 | 3.27 | 1.24 | 11529387 | 6.42 |
| Syringaldehyde | 2.5 | 1.27 | 1.36 | 5684653 | 4.30 |
| Coniferyl aldehyde | 1.25 | 1.40 | 0.41 | 13521998 | 2.04 |
| *p*-Hydroxybenzaldehyde | 1.25 | 1.42 | 0.00 | 15516699 | 1.20 |
| *p*-Hydroxyacetophenone | 1.25 | 1.45 | 0.40 | 15420538 | 0.67 |
| Phenol | 100 | 1.07 | 0.54 | 51494 | 9.13 |
| Guaiacol | 10 | 0.86 | 0.00 | 417310 | 5.30 |
| Coniferyl alcohol | 1.25 | 2.93 | 0.00 | 14462790 | 0.90 |
| p-Hydroxycinnamaldehyde | 0.2 | 1.79 | 0.00 | 10588817 | 2.42 |

**Table S2.**

| **Exp. No** | **Run Order** | **Feed Speed** | **Overfeed Volume** | **Capillary Voltage** | **Gas temperature** | **Gas Flow** | **Sheath Gas Temperature** | **Sheath Gas Flow** |
| --- | --- | --- | --- | --- | --- | --- | --- | --- |
| 1 | 11 | -1 | -1 | -1 | -1 | -1 | -1 | -1 |
| 2 | 10 | 1 | -1 | -1 | -1 | 1 | -1 | 1 |
| 3 | 17 | -1 | 1 | -1 | -1 | 1 | 1 | -1 |
| 4 | 13 | 1 | 1 | -1 | -1 | -1 | 1 | 1 |
| 5 | 2 | -1 | -1 | 1 | -1 | 1 | 1 | 1 |
| 6 | 14 | 1 | -1 | 1 | -1 | -1 | 1 | -1 |
| 7 | 15 | -1 | 1 | 1 | -1 | -1 | -1 | 1 |
| 8 | 12 | 1 | 1 | 1 | -1 | 1 | -1 | -1 |
| 9 | 9 | -1 | -1 | -1 | 1 | -1 | 1 | 1 |
| 10 | 6 | 1 | -1 | -1 | 1 | 1 | 1 | -1 |
| 11 | 18 | -1 | 1 | -1 | 1 | 1 | -1 | 1 |
| 12 | 1 | 1 | 1 | -1 | 1 | -1 | -1 | -1 |
| 13 | 19 | -1 | -1 | 1 | 1 | 1 | -1 | -1 |
| 14 | 3 | 1 | -1 | 1 | 1 | -1 | -1 | 1 |
| 15 | 5 | -1 | 1 | 1 | 1 | -1 | 1 | -1 |
| 16 | 8 | 1 | 1 | 1 | 1 | 1 | 1 | 1 |
| 17 | 16 | 0 | 0 | 0 | 0 | 0 | 0 | 0 |
| 18 | 7 | 0 | 0 | 0 | 0 | 0 | 0 | 0 |
| 19 | 4 | 0 | 0 | 0 | 0 | 0 | 0 | 0 |

**Table S3**

| **Analyte** | **Function** | **OCH3** | **RT** | **Fsp3** | **XLogP** | **MW** | **nAtomP** | **nAtomLC** | **ALogP** | **AMR** | **nAcid** | **tpsaE** | **nHBDon** | **pK_a_** |
| --- | --- | --- | --- | --- | --- | --- | --- | --- | --- | --- | --- | --- | --- | --- |
| *p*-Coumaryl alcohol | VinylAlcohol | 0 | 4.57 | 0.11 | 0.66 | 150.17 | 9 | 9 | 0.66 | 48.94 | 0 | 0.27 | 2 | 9.51 |
| Coniferyl alcohol | VinylAlcohol | 1 | 2.93 | 0.20 | 0.42 | 180.20 | 10 | 9 | 0.17 | 55.58 | 0 | 0.28 | 2 | 9.98 |
| Sinapyl alcohol | VinylAlcohol | 2 | 3.54 | 0.27 | 0.66 | 210.23 | 11 | 9 | -0.33 | 62.22 | 0 | 0.28 | 2 | 9.40 |
| *p*-Hydroxybenzyl alcohol | Alcohol | 0 | 3.84 | 0.14 | -0.12 | 124.14 | 7 | 7 | 0.20 | 38.62 | 0 | 0.33 | 2 | 9.48 |
| Vanillyl alcohol | Alcohol | 1 | 2.43 | 0.25 | -0.15 | 154.16 | 8 | 7 | -0.30 | 45.26 | 0 | 0.32 | 2 | 9.92 |
| Syringol alcohol | Alcohol | 2 | 2.99 | 0.33 | 0.091 | 184.19 | 9 | 7 | -0.80 | 51.91 | 0 | 0.32 | 2 | 9.77 |
| *p-*Hydroxycinnamaldehyde | VinylAldehyde | 0 | 1.79 | 0 | 0.83 | 148.16 | 11 | 9 | 0.92 | 47.95 | 0 | 0.25 | 1 | 9.05 |
| Coniferyl aldehyde | VinylAldehyde | 1 | 1.40 | 0.10 | 0.59 | 178.18 | 12 | 9 | 0.42 | 54.60 | 0 | 0.26 | 1 | 9.52 |
| Sinapinaldehyde | VinylAldehyde | 2 | 1.65 | 0.18 | 0.83 | 208.21 | 13 | 9 | -0.08 | 61.24 | 0 | 0.27 | 1 | 9.67 |
| *p*-Hydroxybenzaldehyde | Aldehyde | 0 | 1.42 | 0 | 0.35 | 122.12 | 9 | 7 | 0.45 | 37.64 | 0 | 0.31 | 1 | 7.32 |
| Vanillin | Aldehyde | 1 | 1.13 | 0.13 | 0.32 | 152.15 | 10 | 7 | -0.05 | 44.28 | 0 | 0.31 | 1 | 7.81 |
| Syringaldehyde | Aldehyde | 2 | 1.27 | 0.22 | 0.56 | 182.17 | 11 | 7 | -0.54 | 50.92 | 0 | 0.31 | 1 | 7.24 |
| *p*-Coumaric acid | VinylAcid | 0 | 4.79 | 0 | 0.75 | 164.16 | 12 | 9 | 0.87 | 48.88 | 1 | 0.35 | 2 | 3.81 |
| Ferulic acid | VinylAcid | 1 | 3.27 | 0.10 | 0.51 | 194.18 | 13 | 9 | 0.37 | 55.52 | 1 | 0.34 | 2 | 3.27 |
| Sinapinic acid | VinylAcid | 2 | 3.78 | 0.18 | 0.75 | 224.21 | 14 | 9 | -0.12 | 62.17 | 1 | 0.34 | 2 | 3.61 |
| *p*-Hydroxybenzoic acid | Acid | 0 | 4.16 | 0 | 0.27 | 138.12 | 10 | 7 | 0.40 | 38.56 | 1 | 0.42 | 2 | 4.38 |
| Vanillic aid | Acid | 1 | 2.74 | 0.13 | 0.24 | 168.15 | 11 | 7 | -0.09 | 45.20 | 1 | 0.40 | 2 | 4.16 |
| Syringic aid | Acid | 2 | 2.97 | 0.22 | 0.48 | 198.17 | 12 | 7 | -0.59 | 51.85 | 1 | 0.38 | 2 | 3.93 |
| *p*-Hydroxyacetophenone | Ketone | 0 | 1.45 | 0.13 | 0.53 | 136.15 | 9 | 7 | 0.39 | 42.11 | 0 | 0.27 | 1 | 7.79 |
| Acetovanillone | Ketone | 1 | 1.10 | 0.22 | 0.50 | 166.17 | 10 | 7 | -0.11 | 48.76 | 0 | 0.28 | 1 | 8.27 |
| Acetosyringone | Ketone | 2 | 1.22 | 0.30 | 0.74 | 196.20 | 11 | 7 | -0.61 | 55.40 | 0 | 0.28 | 1 | 7.71 |
| Phenol | Phenol | 0 | 1.07 | 0 | 0.95 | 94.11 | 7 | 5 | 0.84 | 32.56 | 0 | 0.22 | 1 | 10.0 |
| Guaiacol | Phenol | 1 | 0.86 | 0.14 | 0.71 | 124.14 | 8 | 6 | 0.34 | 39.20 | 0 | 0.24 | 1 | 9.98 |
| Syringol | Phenol | 2 | 0.94 | 0.25 | 0.95 | 154.16 | 9 | 7 | -0.16 | 45.85 | 0 | 0.25 | 1 | 9.37 |

**Figure S1**
